# Iron and Advanced Glycation End Products: Emerging Role of Iron in Androgen Deficiency in Obesity

**DOI:** 10.3390/antiox9030261

**Published:** 2020-03-22

**Authors:** Seu-Hwa Chen, Kuo-Ching Yuan, Yu-Chieh Lee, Chun-Kuang Shih, Sung-Hui Tseng, Alexey A. Tinkov, Anatoly V. Skalny, Jung-Su Chang

**Affiliations:** 1 Department of Anatomy and Cell Biology, School of Medicine, College of Medicine, Taipei Medical University, Taipei 110, Taiwan; seuhwa@tmu.edu.tw; 2 Department of Emergency and Critical Care Medicine, Taipei Medical University Hospital, Taipei 110, Taiwan; traumayuan@gmail.com; 3 Department of Obstetrics and Gynecology, Taipei Medical University Hospital, Taipei 110, Taiwan; julyeast@gmail.com; 4 School of Nutrition and Health Sciences, College of Nutrition, Taipei Medical University, Taipei 110, Taiwan; ckshih@tmu.edu.tw; 5 Department of Physical Medicine and Rehabilitation, Taipei Medical University Hospital, Taipei 110, Taiwan; m003089010@tmu.edu.tw; 6 Department of Physical Medicine and Rehabilitation, School of Medicine, College of Medicine, Taipei Medical University, Taipei 110, Taiwan; 7 Department of Medical Elementology, Peoples’ Friendship University of Russia (RUDN University), Moscow 117198, Russia; tinkov.a.a@gmail.com (A.A.T.); skalnylab@gmail.com (A.V.S.); 8 Laboratory of Biotechnology and Applied Bioelementology, Yaroslavl State University, Yaroslavl 150003, Russia; 9 Laboratory of Molecular Dietology, IM Sechenov First Moscow State Medical University, Moscow 119146, Russia; 10 Graduate Institute of Metabolism and Obesity Sciences, College of Nutrition, Taipei Medical University, Taipei 110, Taiwan; 11 Nutrition Research Center, Taipei Medical University Hospital, Taipei 110, Taiwan; 12 Chinese Taipei Society for the Study of Obesity (CTSSO), Taipei 110, Taiwan

**Keywords:** obesity, iron, fat, advanced glycation end products, testis, testosterone

## Abstract

The literature suggests a bidirectional relationship between testosterone (T) and iron, but mechanisms underlying this relationship remain unclear. We investigated effects of iron on advanced glycation end products (AGEs) in obesity-related androgen deficiency. In total, 111 men were recruited, and iron biomarkers and N(*ɛ*)-(carboxymethyl)lysine (CML) were measured. In an animal study, rats were fed a 50% high-fat diet (HFD) with (0.25, 1, and 2 g ferric iron/kg diet) or without ferric citrate for 12 weeks. Obese rats supplemented with >1 g iron/kg diet had decreased testicular total T compared to HFD alone. Immunohistochemical staining showed that >1 g of ferric iron increased iron and AGE retention in testicular interstitial tissues, which is associated with increased expression of the receptor for AGEs (RAGE), tumor necrosis factor-α, and nitric oxide. Compared with normal weight, overweight/obese men had lower T levels and higher rates of hypogonadism (19% vs. 11.3%) and iron overload (29.8% vs.15.9%). A correlation analysis showed serum total T was positively correlated with transferrin saturation (*r* = 0.242, *p* = 0.007) and cathepsin D (*r* = 0.330, *p* = 0.001), but negatively correlated with red blood cell aggregation (*r* = −0.419, *p*<0.0001) and CML (*r* = −0.209, *p* < 0.05). In conclusion, AGEs may partially explain the underlying relationship between dysregulated iron and T deficiency.

## 1. Introduction

Numerous studies have documented that androgen deficiencies and dysregulation of iron homeostasis are common features observed in obese men [1,2,3]. In Taiwan, one in two men is either overweight or obese [4], and one-fourth of Taiwanese men have an androgen deficiency (24.1%) (total testosterone (T) of <300 ng/dL or free T of <5 ng/dL) [5] and iron overload (21.5%) (serum ferritin>300ng/mL) [6,7]. There is a bidirectional relationship between iron metabolism and androgen synthesis, and obesity may affect this relationship. It has been known for more than half a century that T, the male sex hormone, is involved in erythropoiesis [8]. Low T levels are associated with the development of anemia in the elderly [9] and men with type 2 diabetes [10]. Recent clinical studies showed that T administration increase iron absorption and red blood cells (RBCs) synthesis through the suppression of the master iron regulator hepcidin hormone [11,12,13]. Hepcidin is a liver-synthesized hormone that regulates iron absorption and mobilization. However, obesity-related inflammation triggers abnormal hepcidin synthesis leading to disruption of iron homeostasis and tissue iron overload [14]. As the human body lacks a mechanism for eliminating iron, tissue iron overload is known to exert pathophysiological effects on androgen synthesis [15]. Elevated serum ferritin are common feature observed in obese men and serum ferritin levels are negatively correlated with serum total T levels in adult men and young adolescent boys [16,17]. A pituitary iron overload predicts hypogonadism in thalassemia patients with transfusional iron overload [18]. Gauiter et al. [19] studied 50 men with a moderate dysmetabolic iron excess and found that 8% had low total T (<10.4 nmol/L) and 26% had low bioavailable T (<2.5 nmol/L), suggesting that liver iron overload is associated with moderate hypogonadotropic hypogonadism. Twelve weeks of iron therapy (800~1200 mg elemental Fe) increased total T and sperm parameters in eugonadal men with iron-deficiency anemia (IDA) [20]. Overall, these data suggest that the interplay between iron and T are of clinical importance and obesity-related inflammation may disrupt this intricate balance. 

There is a growing body of evidence implicating advanced glycation end products (AGEs) and the receptor of AGEs (RAGE) in the progression of obesity-related complications such as androgen deficiency [21] and altered reproductive function [22]. AGEs are a diverse group of compounds, which are synthesized from non-enzymatic reactions of a reducing sugar or oxidized lipid with amino acids [23]. The modern diet contains high amount of AGEs, particular in dry heat-processed foods [24]. Although dietary AGEs and serum AGE levels are associated with chronic diseases [21,22,25,26,27,28], outcomes of human studies are sometimes inconclusive [24]. For example, AGEs also form endogenously, particularly in patients with diabetes [25]. The ability of the body to detoxify modified AGE proteins also plays an important role [23]. Intracellular AGEs (derived from endogenous or exogenous sources) can interact with family of AGE receptors (AGERs) but they seem to play distinct roles in AGE toxicity or detoxification [29]. The ligation of AGEs to the RAGE triggers oxidative responses and cytokine cascade through the phosphorylation of mitogen-activated protein kinase p38 (p38 MAPK) and the activation of NFκB [26]. Cluster of differentiation 36 (CD36) can mediate the uptake of AGEs through endocytosis, but it also acts as a fatty acid translocase and can mediate the uptake of fatty acids or lipids. Lipids like low-density lipoprotein are sensitive to AGE cross-linking which may cause arterial stiffness [30]. On the other hand, AGER1 (OST-48) was shown to play an important role in the clearance of AGE-modified protein [26]. Early cell culture studies showed that AGEs inhibited AGER1 expression [31] and overexpression of AGER1 inhibited AGE-RAGE activation [29]. AGEs-modified protein can be removed by scavenger receptors through endocytosis and degraded by enzymes that located in lysosomes (e.g., cathepsin D (CTSD) or the cytoplasm [e.g., the glutathione-dependent glyoxalase 1 (Glo1) enzymatic defense] [23]. The soluble extracellular domain of RAGE (sRAGE) is able to act as a decoy to avoid the ligation of AGEs to RAGE [25]. An inability to remove intracellular AGEs leads to ligation of AGEs, or major toxic derivatives such as N(*ɛ*)-(carboxymethyl)lysine (CML), to RAGE, resulting in robust proinflammatory and oxidative cellular responses [26].

A bidirectional relationship between iron and AGE metabolism may also exit. Early studies showed that Maillard reaction products (MRP) affects iron bioavailability [32] as MRPs act as metal chelators [33]. In addition, rats fed with MRP-rich diet for 88 days had decreased levels of hemoglobin but increased iron retention in the liver, suggesting a direct effect of MRP on intracellular iron mobilization [34]. These findings are consistence with human studies which showed an inverse relationship between serum CML and anemia, but the causal relationship between increased CML levels and anemia remains undefined [35,36]. Very few studies investigated the effects of iron on AGEs metabolism. In 2018, Mirlohi et al. [37] first reported that plasma levels of CML and pentosidine (PTD) were increased in the patients with β-thalassemia major, and serum PTD, not CML, were positively correlated with markers of iron overload (serum ferritin, iron). Early in vitro studies showed that CML is formed during copper-catalyzed oxidation of polyunsaturated fatty acids in the presence of protein [38] and the formation of CML is suppressed by the addition of iron chelator desferrioxamine or iron-containing ant-oxidative enzymes such as catalase and superoxide dismutase (SOD2) [39]. Our previous study showed that streptozotocyin/nicotinamide-induced diabetic rats received >1g ferric iron supplementation had increased liver AGEs concentrations and iron-mediated lipid peroxidation may interfere with AGEs metabolism [27]. In this study, we tested the hypothesis that excess iron may interfere with AGEs metabolism, and the dual effects of iron-AGEs may affect male sex hormone T levels in obese men. To this end, we recruited 111 adult men (44 of normal weight and 67 overweight (OW) or obese) and conducted a 12-week feeding experiment in rats with high-fat diet (HFD)-induced obesity supplemented without (37 mg ferric iron/kg diet) or with ferric citrate (0.25, 1, and 2 g ferric iron/kg diet).

## 2. Materials and Methods

### 2.1. Human Study

Adult men, aged 20~65 years, were recruited when they visited the Clinic of Gastroenterology and Hepatobiliary Disease at Taipei Medical University Hospital (Taipei, Taiwan) in July 2015 to June 2016. Exclusion criteria were a history of liver diseases (e.g., hepatitis virus infection, drug-induced hepatitis), alcohol intake of >30 g/week, and other chronic diseases such as cancer, nephritis, or autoimmune disease. Inclusion criteria were age≥19 years old and with Taiwanese passport. In total, 111 adult men [44 normal weight and 67 overweight (OW) and obese] aged 41.06 ± 11.51 years were recruited for this study. Serum total T and sex hormone-binding protein (SHBG) were detected with a electrochemiluminescence immunoassay (Cobas E601, Roche Diagnostic, Mannheim, Germany). Serum iron and the total iron-binding capacity (TIBC) were measured using a ferrozine-based colorimetric method. The % transferrin saturation (%TS) was calculated as (serum iron/TIBC) × 100. Serum CTSD (Cloud-Clone, Wuhan, Hubei Province, China), sRAGE (extracellular domain) (R&D System, Minneapolis, MN, USA) and free Hb (ICL, Portland, OR, USA), were assessed by an enzyme-linked immunosorbent assay (ELISA) according to the manufacturer’s instructions. For RBC aggregation, 500 μL of whole blood was added to a disposable plastic chip (RSD-K02), and RBC aggregation was evaluated using a RheoScan-AnD300 analyzer (MicroStar Instruments, Korea). The serum malondialdehyde (MDA) concentration, a lipid peroxidation marker, was determined based on the reaction of MDA with thiobarbituric acid to form thiobarbituric acid-reactive species. Serum CML were analyzed by an ELISA, according to the manufacturer’s instructions (Cell Biolabs, San Diego, CA, USA). Iron overload was defined as adult men with serum ferritin >300 ng/mL [7]. Hypogonadism was defined as serum total T of<3 ng/mL [40]. This study was approved by the Taipei Medical University Institutional Ethical Review Committee (TMU-JIRB 201502018), and written informed consent was obtained from all participants.

### 2.2. Animal Study

Thirty (*n* = 6/group) male Sprague-Dawley (SD) rats aged 8 weeks were randomly assigned to five groups: (1) a control group fed a normal diet with 36.7 mg ferric iron/kg diet (14.9% protein, 9.4% fat, 75.7% carbohydrates; total energy: 3.81 kcal/g diet), (2) an HFD group with 36.7 mg ferric iron/kg diet (14.8% protein, 50.0% fat, 38.7% carbohydrates; total energy: 4.80 kcal/g diet), (3) an HFD group plus 0.25 g ferric iron/kg diet (14.8% protein, 50.0% fat, 38.7% carbohydrates; total energy: 4.80 kcal/g diet), (4) an HFD group plus 1 g ferric iron/kg diet (14.8% protein, 50.0% fat, 38.7% carbohydrates; total energy: 4.80 kcal/g diet), and (5) an HFD group plus 2 g ferric iron/kg diet (14.8% protein, 50.0% fat, 38.7% carbohydrates; total energy: 4.80 kcal/g diet) for 12 weeks. All animal procedures were conducted under a protocol approved by the Animal Ethical Committee of Taipei Medical University (LAC-2014-0254). Ferric citrate was purchased from Sigma-Aldrich (St. Louis, MO, USA). The AIN-93 M standard diet was purchased from MP Biomedicals (Fisher Scientific; New Taipei City, Taiwan).

### 2.3. Serum and Tissue Lysate Biochemical Measurements

Fasting blood samples were collected from overnight-fasted SD rats. Serum and plasma were stored at −80 °C until being analyzed. To measure tissue levels of iron, CML, total T, MDA, nitric oxide (NO), and tumor necrosis factor (TNF)-α, 25~50-mg tissue samples were homogenized in 200 μL of iron assay buffer or 300 μL of RIPA lysis buffer (Merck Millipore, Taipei, Taiwan), using a TissueLyser II (Qiagen, Hilden, Germany). The total protein content was determined by the Bradford method (Carl Roth, Karlsruhe, Germany). Serum and testicular total T concentrations were determined with a total T enzyme-linked immunosorbent assay (ELISA) kit (Cayman Chemical, Ann Arbor, MI, USA). Testicular TNF-α concentrations were measured with a rat TNF-α ELISA kit (eBioscience, San Diego, CA, USA). Serum and testicular concentrations of *malondialdehyde* (MDA), a lipid peroxidation marker, were determined based on the reaction of MDA with thiobarbituric acid to form *thiobarbituric acid*-reactive species. Serum, testis, and liver CML and AGEs levels were analyzed by an ELISA, according to the manufacturer’s instructions (Cell Biolabs, San Diego, CA, USA). For the CML quantification, the IgG was depleted from each sample prior to testing by protein A antibody purification kit (BioVision, Milpitas, CA, USA). Testicular iron was detected by staining with Perl’s Prussian blue iron (ScY Tek Laboratories, Logan, UT, USA) or with an iron assay kit (Abcam, Cambridge, UK). As a biomarker of NO synthesis, testicular NO levels were measured by the Griess reagent (Panreac Quimica, Taipei, Taiwan).

### 2.4. Immunohistochemical (IHC) Staining

Fresh liver and testis samples were placed in 10% buffered formalin and subsequently embedded in paraffin. IHC was performed on 5-µm-thick formalin-fixed paraffin-embedded liver and testicular sections using an Ultravision Quanto Detection System HRP Polymer kit (ThermoFisher Scientific, Fremont, CA, USA). Briefly, slides were deparaffinized and hydrated. Deparaffinization was performed in xylene (Histolab, Gothenburg, Sweden) twice for 15 min, followed by hydration in a graded alcohol series, including blocking of endogenous horseradish peroxidase (HRP) activity for 10 min. Non-specific proteins were blocked with Ultra V Block reagents. Primary antibodies (Abs) [(AGE (1:100 for testis and 1:1000 for liver) (Merck, Darmstadt, Germany) and RAGE (1;200) (R&D, Minneapolis, MN, USA)] were applied to slides and incubated for 24 h at 4 °C. The next day, primary Abs were removed by a washing step. A biotinylated goat secondary anti-mouse Ab [AGE (1:50) and RAGE (1:20)] was applied to the slides (Vector, Burlingame, CA, USA), and slides were incubated for 2 h at 37 °C. After any unbound secondary Abs were washed off, an avidin/biotinylated enzyme complex (ABC) (1:200) was added, and slides were incubated for 2 h at 37 °C. Slides were stained with substrates of diaminobenzidine (DAB) or Vector SG (both purchased from Vector Laboratories, Taipei, Taiwan) and mounted. All tissues were scanned using an automated slide-scanning system with an automated immunostainer (Ventana Discovery XT Autostainer, USA). Expressions of tissue iron, AGE, and RAGE are presented as the number of positive stained cells per 10^5^ μm^2^ estimated by a TissueGnostics Tissue FAXS and HistoFAXS Analysis system (TissueGnostics, Vienna, Austria). Briefly, numbers of Perl’s Prussian blue iron-stained, and AGE and RAGE positively stained cells were randomly counted in nine areas per tissue slide with each area containing 0.2092 mm^2^ (1753 × 1753 pixels/area). For each slide, the mean number of positively stained cells was averaged from the nine areas. The total number of positively stained cells is presented as the mean positively stained cells from three tissue slides per treatment group for AGE and RAGE (*n* = 3/group) and four tissue slides per treatment group (*n* = 4/group) for Perl’s Prussian blue iron staining.

### 2.5. Western Blot Analysis

Liver and testicular tissues at 25~50 mg were lysed in RIPA buffer, and the protein concentration was determined with a Pierce™ BCA Protein Assay Kit (Thermo Fisher Scientific, Waltham, MA, USA). Anti-rat Abs for tubulin, AGER1,cluster of differentiation 36 (CD36), GLO-1, p38 mitogen-activated protein kinase (p38MAPK), and RAGE were used at a dilution of 1:1000 to detect immunoreactive signals, except for CML (1:100), tubulin (1:5000), and CTSD (1:2000). Abs were purchased from Santa Cruz Biotechnology (Santa Cruz, San Jose, CA, USA), except for GLO-1 (Abcam, Cambridge, UK), RAGE (Abcam), CML (Abcam), tubulin (GeneTex, Hsinchu *City*, Taiwan), and CTSD (Sigma-Aldrich, Taipei, Taiwan). Blots were visualized using an enhanced chemiluminescence (ECL)-Plus detection kit (PerkinElmer Life Sciences, Boston, MA, USA).

### 2.6. Statistical Analysis

Statistical analyses were conducted using SPSS 19 (IBM, Armonk, NY, USA) and GraphPad Prism 4 (La Jolla, San Diego, CA, USA). For the rat study, a one-way analysis of variance (ANOVA) with the Bonferroni posttest correction was used to analyze differences between groups [*n* = 6/group for body weight (BW) and biochemistry; *n* = 3~4/group for Western blotting, AGE and RAGE IHC, and Perl’s Prussian blue iron staining]. A linear trend testing of continuous data was analyzed by a linear regression. Correlations of testicular total T, CML, TNF-α, and AGE with iron positively stained cells were evaluated using Spearman rank-order correlation coefficients (n = 3~6/group). A repeated-measures ANOVA was used for the time course analysis of changes in BW. For the human study, data are presented as the mean ± standard deviation (SD). Associations between the serum total T concentration and other serum biochemistry parameters were evaluated using Spearman’s rank correlations coefficients and multivariate linear regression analysis. The Mann–Whitney U test was employed for the two group comparison. *p* < 0.05 was considered statistically significant.

## 3. Results

### 3.1. Ferric Citrate Supplementation Decreases Testicular Total T Levels and Increases Serum MDA in Obese Male Rats

**Figure 1:**
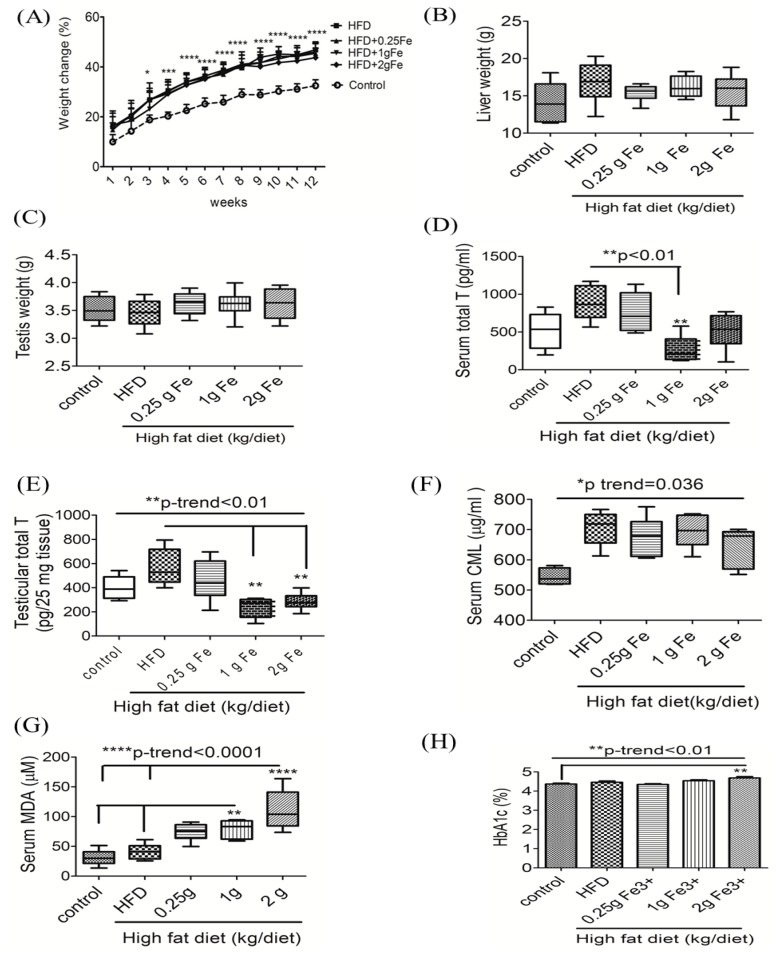
Effects of ferric citrate supplementation on the body weight, organ weight, and serum biochemistry. Percentage (%) changes in body weight (**A**), liver weight (**B**), testis weight (**C**), serum total testosterone (T) (**D**), testicular total T (**E**), serum N(epsilon)-(carboxymethyl)lysine (CML) (**F**), serum malondialdehyde (MDA) (**G**) and HbA1c (**H**) in lean and obese rats after 12 weeks of feeding a high-fat diet (HFD), HFD plus ferric iron supplementation, or a standard diet. Data are expressed as the mean ± SEM (*n* = 6/group). * *p* < 0.05; ** *p* < 0.01; *** *p* < 0.001; **** *p* < 0.0001 vs. the controls or the HFD by a one-way ANOVA with the Bonferroni posttest and correction. A repeated-measures ANOVA was used for the time course analysis of changes in body weight (**A**). The trend test of continuous data was analyzed by a linear regression.

In the first week after switching from the standard diet to the iron/fat-enriched diet, ~50% of the food remained uneaten in the 1- and 2-g iron groups. Most rats gradually accepted the iron/fat-enriched diet after 7 days of feeding, but rats in the 2-g iron group tended to eat less than those in the other groups. Figure 1A shows that rats which received 12 weeks of an HFD had gained on average >20% of their initial BW. In spite of the weight gain, no difference was observed in the liver weight (Figure 1B) or testicular weight (Figure 1C) between lean and obese rats. Although a trend toward decreased serum total T was apparent in the 1- and 2-g ferric iron groups compared to the HFD alone group, only the 1-g ferric iron group reached statistical significant difference (*p* < 0.01), while the 2-g iron group did not due to high group variation (Figure 1D). However, both 1 and 2 g of ferric iron caused a significant reduction in testicular total T levels compared to the HFD-only group (all *p* < 0.01) (Figure 1E). A small linear trend towards increased serum CML levels was observed in obese rats, suggesting that iron supplementation did not influence circulating CML levels (*p* for trend <0.05) (Figure 1F). Although iron supplementation did not increase serum CML levels, high doses of ferric iron significantly increased serum MDA (Figure 1G) and HbA1c levels (Figure 1H) (all *p* < 0.05).

### 3.2. Impacts of Ferric Citrate Supplementation on the Hepatic AGE-RAGE Axis

**Figure 2:**
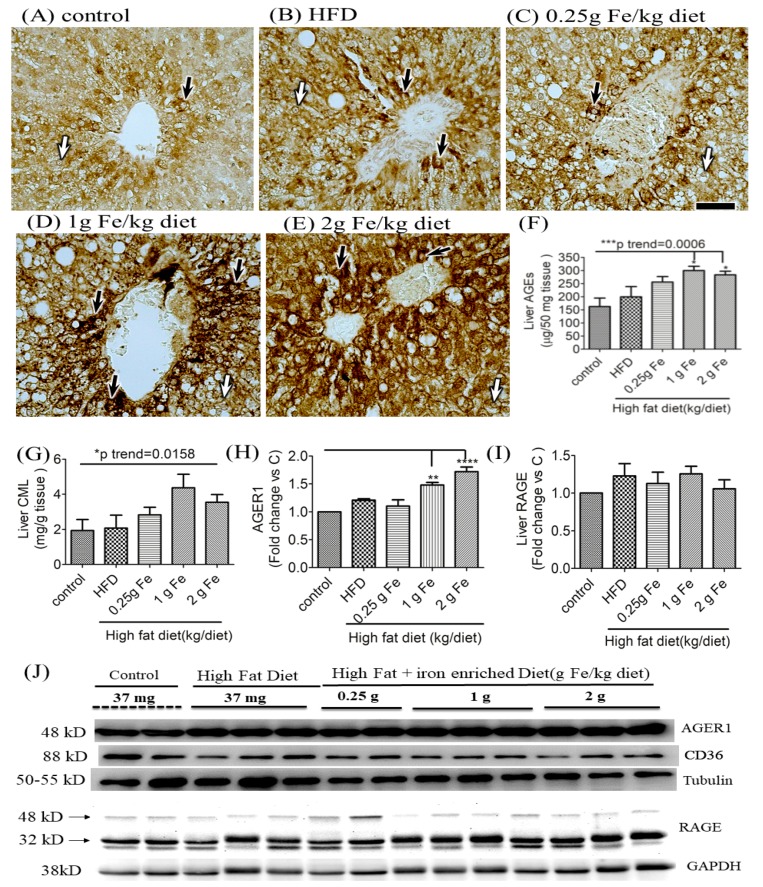
Effects of ferric citrate supplementation on liver advanced glycation end products (AGEs). Liver AGEs were detected by IHC staining in all groups (**A**–**E**) (Scale bar 50 μm). Black arrow indicates AGEs-immunoreactive positive hepatocyte and white arrow indicates AGEs-immunoreactive negative hepatocyte. Expression of liver AGEs (**F**) and N(epsilon)-(carboxymethyl)lysine (CML) (**G**) was quantitated with an ELISA kit. Hepatic expressions of AGE receptor 1 (AGER1) (**H**,**J**), receptor of AGEs (RAGE) (**I**,**J**), and CD36 (**J**) were detected by a Western blot analysis and normalized against tubulin (**J**) or GAPDH (**J**) (*n* = 3 or 4/group). Data are expressed as the mean ± SEM. * *p* < 0.05; ** *p* < 0.01; *** *p* < 0.001; vs. the controls by a one-way ANOVA with the Bonferroni posttest and correction. The trend test of continuous data was analyzed by a linear regression.

IHC staining of AGEs in liver tissue samples showed positive staining of AGEs (+) in all rats, but obese rats fed with >1-g ferric iron were appeared to be higher than those of other groups (Figure 2A–E). We next quantitated hepatic concentrations of AGEs, and CML, the major immunogen of AGEs. Analysis of hepatic AGEs lysates showed, compared with control, >1g ferric iron significantly induced AGEs expression (Figure 2F, *p* for trend = 0.0006). A similar trend was observed in CML levels in liver lysates, but to a lesser extent (Figure 2G, *p* for trend = 0.0158). A Western blot analysis also revealed that >1 g of ferric iron supplementation triggered hepatic AGER1 protein expression (Figure 2G,J, all *p* < 0.01), but no difference was found in expressions of CD36 (Figure 2H,J) or RAGE (Figure 2I,J); the latter was confirmed by IHC staining (data not shown). Inactivation of hepatic RAGE expression indicates that the liver was able to detoxify and clear AGEs.

### 3.3. Impacts of Ferric Citrate Supplementation on the Testicular AGE-RAGE Axis

**Figure 3:**
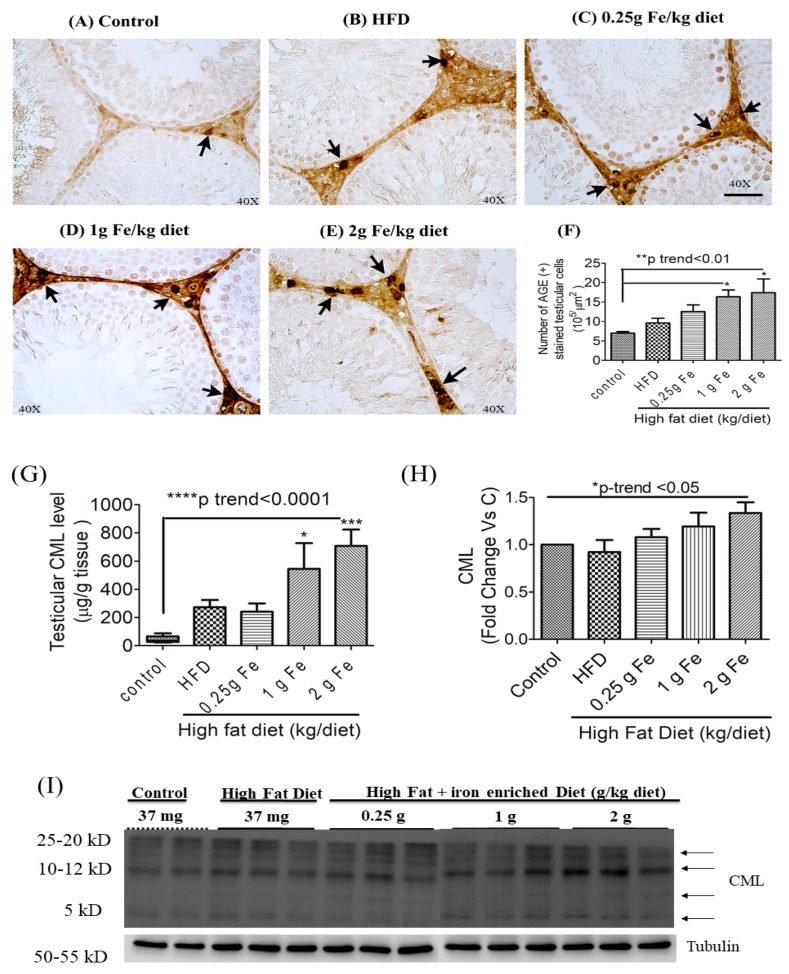

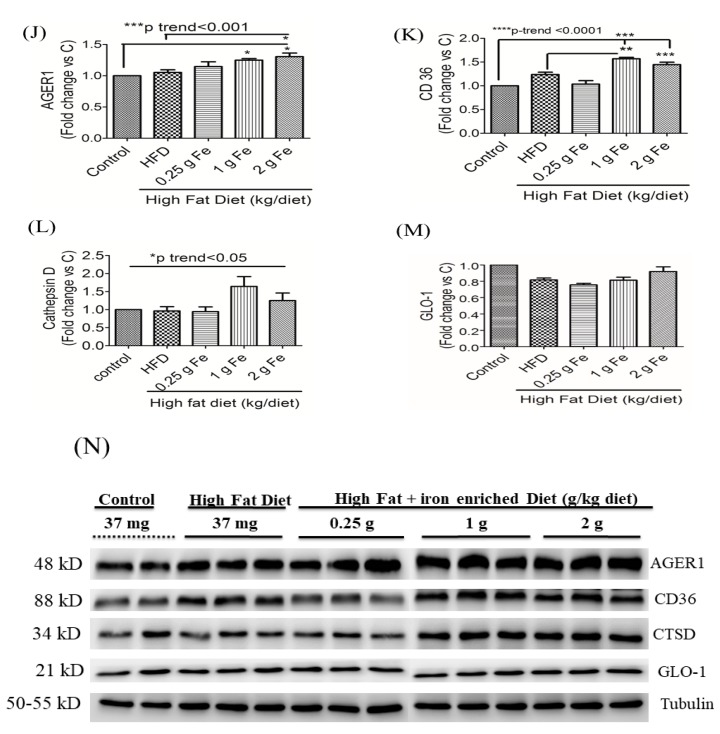
Effects of ferric citrate supplementation on testicular advanced glycation end products (AGEs). Testicular AGEs (black arrow) (**A**–**F**) were detected by IHC staining (*n* = 3/group) (Scale bar 50 μm). Expression of testicular N(epsilon)-(carboxymethyl)lysine (CML) was quantitated by an ELISA (**G**) (*n* = 6/group) and Western blotting (**H**,**I**). Expressions of testicular AGE receptor 1 (AGER1) (**J**,**N**), CD36 (**K**,**N**), cathepsin D (CTSD) (**L**,**N**), and glutathione-dependent glyoxalase (GLO-1) (**M**,**N**) were detected by a Western blot analysis (*n* = 3 or 4/group). Data are expressed as the mean ± SEM. * *p* < 0.05; ** *p* < 0.01; *** *p* < 0.001 vs. the control or high-fat diet (HFD) by a one-way ANOVA with the Bonferroni posttest and correction. The trend test of continuous data was analyzed by a linear regression.

IHC staining of AGEs in testicular tissue samples showed that >1 g of ferric iron increased the number of AGE (+) positively stained cells (Figure 3A–F). Quantification of concentrations of CML by an ELISA (G) or by Western blotting (H and I) also revealed that compared to the control or HFD alone, high doses of ferric iron significantly increased CML expression in the testes (all *p* < 0.05) (Figure 3). The Western blot analysis also revealed that, compared to rats fed the standard diet or HFD, >1 g of ferric iron increased the expression of AGE and degradation proteins, AGER1 (Figure 3J,N), CD36 (Figure 3K,N), and, to a lesser extent, CTSD (Figure 3L,N), but not GLO-1 (Figure 3M,N). 

**Figure 4:**
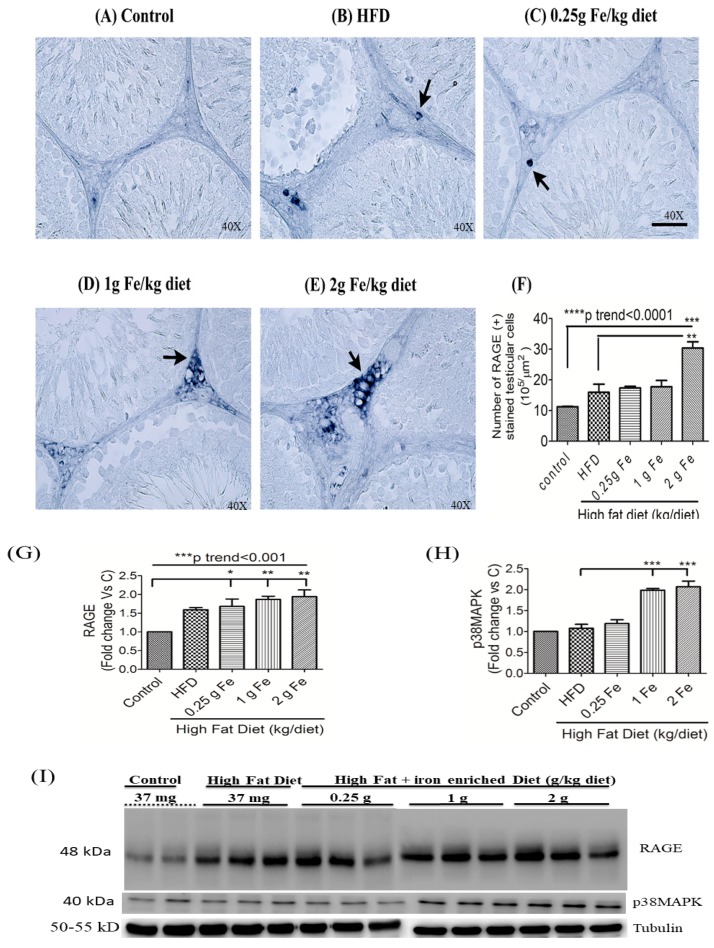
Effects of ferric citrate supplementation on the testicular receptor for advanced glycation end products (RAGE). Testicular RAGE (**A**–**F**) was detected by IHC staining (**A**–**F**) (*n* = 3/group) and a Western blot analysis (**G**,**J**). Expressions of testicular RAGE (**G**,**I**) and p38MAPK (**H**,**I**) were detected by a Western blot analysis. Western blot data are expressed as the mean ± SEM (*n* = 3 or 4/group). * *p* < 0.05; ** *p* < 0.01; *** *p* < 0.001; **** *p* < 0.0001 vs. the controls or high-fat diet (HFD) by a one-way ANOVA with the Bonferroni posttest and correction. The trend test of continuous data was analyzed by a linear regression.

Micrographic IHC staining of testicular RAGE showed that 2 g of ferric iron induced a significantly higher number of RAGE (+)-stained cells (*p* < 0.01; *p* for trend <0.0001) (Figure 4A–F). A Western blot analysis of testicular lysates confirmed that ferric iron induced RAGE (Figure 4G,I) and p38MAPK expressions (Figure 4H,I) in obese rats.

### 3.4. Impacts of Ferric Citrate Supplementation on Testicular Iron, TNF-α, NO, and MDA Levels

**Figure 5:**
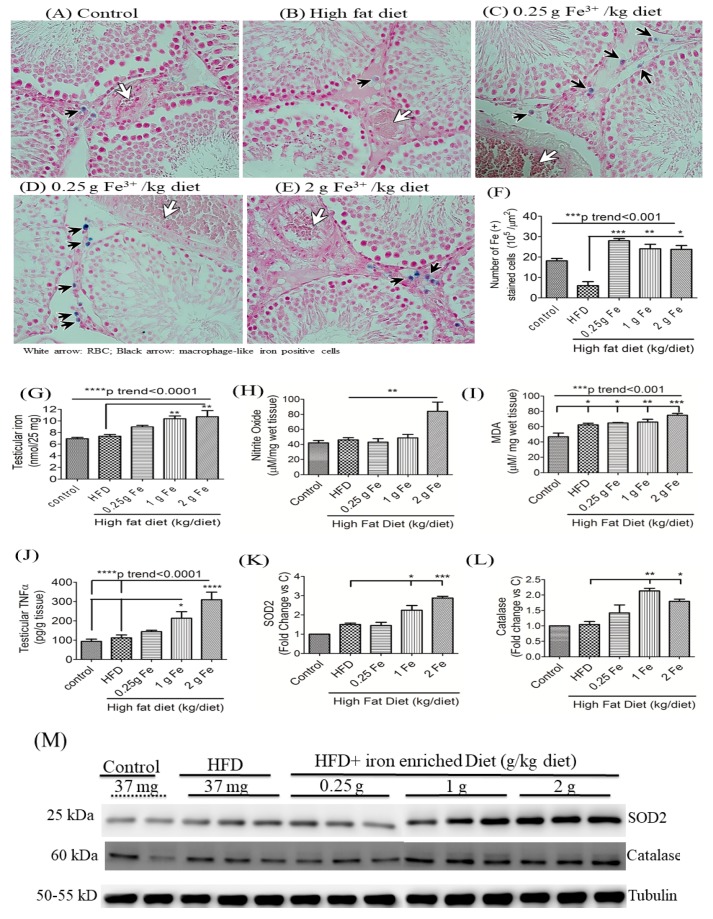
Effects of ferric citrate supplementation on testicular iron, tumor necrosis factor (TNF)-α, oxidants, and antioxidants in rats with high-fat diet (HFD)-induced obesity. Perl’s Prussian blue iron staining (**A**–**F**) showed deep-blue iron-stained granules in macrophage-like cells of interstitial tissues (black arrow) in groups fed the HFD plus ferric citrate supplementation in testes (magnification 400×). The white arrow indicates red blood cells, and the black arrow indicates macrophage-like iron positively stained cells (*n* = 4/group) (**A**–**E**). Testicular total iron (**G**), nitrite oxide (NO) (**H**), malondialdehyde (MDA) (**I**), and TNF-α (**J**) were detected by an ELISA. Antioxidant proteins superoxide dismutase 2 (SOD2) (**K**,**M**) and catalase (**L**,**M**) were detected by a Western blot analysis (*n* = 3 or 4/group). * *p* < 0.05; ** *p* < 0.01; *** *p* < 0.001; **** *p* < 0.0001 vs. the HFD by a one-way ANOVA with the Bonferroni posttest and correction. The trend test of continuous data was analyzed by a linear regression.

Perl’s Prussian blue iron staining showed deep-blue iron-stained (+) granules in macrophage-like cells of interstitial tissues in the testes (black arrow) of groups fed the HFD with ferric iron supplementation (Figure 5A–F) (all *p* < 0.05). Iron quantification results confirmed that obese rats that received >1 g of iron supplementation had higher testicular total iron levels compared to rats that only received the HFD (Figure 5G) (all *p* < 0.01; *p* for trend <0.0001). Furthermore, high doses of ferric iron supplementation also significantly increased testicular NO (Figure 5H), MDA (Figure 5I), and TNF-α levels (Figure 5J) compared to the standard diet or HFD alone (all *p* < 0.05). A Western blot analysis of testicular lysates found that >1 g of the ferric iron induced expressions of the antioxidants, superoxide dismutase 2 (SOD2; Figure 5K,M) and catalase (Figure 5L,M), in obese rats compared to the HFD alone (all *p* < 0.05).

### 3.5. Correlations among Testicular Total T, AGEs, and Iron

**Figure 6:**
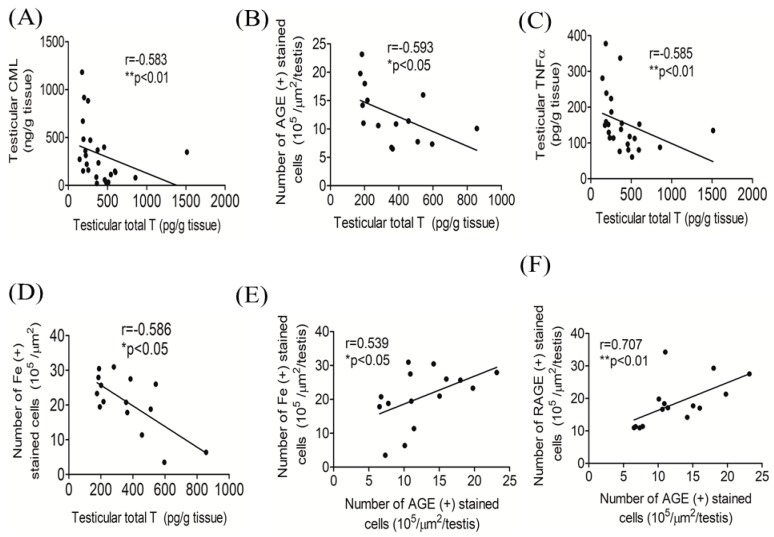
Spearman’s rank correlation analysis of testicular total testosterone (T) and N(epsilon)-(carboxymethyl)lysine (CML) (*n* = 5/group) (**A**), testicular total T, and the number of advanced glycation end products (AGE)-positive cells (*n* = 3/group) (**B**), testicular total T and tumor necrosis factor (TNF)-α (*n* = 5/group) (**C**), and testicular total T and number of iron positively stained cells (*n* = 3/group) (**D**). Associations between the number of AGE positively stained cells and the number of iron positively stained cells in testis (*n* = 3/group) (**E**), and the number of RAGE positively stained testicular cells (*n* = 3/group) (**F**). * *p* < 0.05; ** *p* < 0.01.

A Spearman’s correlation analysis showed that testicular total T levels were inversely correlated with testicular CML (*r* = −0.583, *p* < 0.01) (Figure 6A), the number of AGE positively stained testicular cells (*r* = −0.593, *p* < 0.05) (Figure 6B), testicular TNF-α (*r* = −0.585, *p* < 0.01) (Figure 6C), and the number of testicular iron positively stained cells (*r* = −0.586, *p* < 0.05) (Figure 6D). Significant positive correlations were also observed between the number of testicular AGE (+)-stained and iron (+)-stained cells (*r* = 0.539, *p* < 0.05) (Figure 6E) as well as the number of testicular RAGE (+)-stained cells (*r* = 0.707, *p* < 0.001) (Figure 6F).

### 3.6. Human Study: Correlations among Serum Total T, Iron and AGEs Biomarkers

We next investigated relationship among iron, AGEs, and the male sex hormone T in 111 adult men. 

Table 1 shows that OW/Obese adult men had lower total T levels and higher prevalence rates of hypogonadism (19% vs. 11.3%) and iron overload (29.8% vs. 15.9%) (also known as hyperferritimia) compared with normal weight men. Serum biochemistry analysis also found that, compared with normal weight men, OW/Obese men had higher serum CML, serum free Hb, ferritin, and RBC aggregation but lower serum CTSD and total T levels (all *p* < 0.05).

Spearman’s correlation coefficient analysis showed serum total T was positively correlated with %TS (*r* = 0.242, *p* = 0.007) and CTSD (*r* = 0.330, *p* = 0.001) but negatively correlated with RBC aggregation (*r* = -0.419, *p* < 0.0001) and CML (*r* = −0.209, *p* < 0.05) (Table 2). Serum CML levels were negatively correlated with free Hb (*r* = −0.365, *p* = 0.0004) but positively correlated with sRAGE (*r* = 0.213, *p* = 0.04) (Table 2). A weak positive relationship between serum iron and MDA levels (*r* = 0.2143, *p* = 0.017) was also observed (data not shown). The influence of iron and AGE on total T level was confirmed by the multivariate linear regression analysis, which showed that, after being adjusted with age and BMI, CTSD (ß = 0.0002 (0.00003~0.00036), *p* = 0.018) and RBC aggregation (ß = −0.006 (−0.010~−0.002), *p* = 0.001) remained significant predictors of total T (data not shown). 

## 4. Discussion

Obese men are commonly associated with androgen deficiency and iron dysregulation. Using human and diet-induced obese rat models, we demonstrated for the first time that an obesity-related androgen deficiency can partially be explained by the dual effects of iron and AGEs on T synthesis. There are several interesting finding in this study, which may help to shed some light on how iron dysregulation affect male reproduction function. Firstly, human study suggests that serum free Hb and CTSD, a lysosomal protease, may act as interplay for iron-AGE-T relationship. Our human study found that, compared to normal weight, OW/obese men had higher free Hb, RBC aggregation, and CML levels but lower serum CTSD levels, and serum CTSD and RBC aggregation were the independent predictor of total T levels. CTSD is a lysosomal protease which has numerous biological function including protein degradation (e.g., globin chain of Hb and AGE-modified protein) and hormone processing. As a long-lived protein, RBC is prone for glycation. Glycation of RBC protein may alter RBC rheological property (e.g., aggregability or deformability) resulting in Hb bursts from RBC [41]. Serum free Hb or AGE-modified protein can be uptake through receptor-mediated endocytosis or phagocytosis and degrade by protease through endosomal-lysosomal system [42,43]. The current data suggests that over-nutrition (e.g., fat, iron) or increased toxin (AGEs) may cause lysosomal stress and circulating CTSD levels may serve as a metabolic intermediator to reflect the intricate balance between iron-AGEs and male sex hormone. Secondly, we found strong inverse relationship between serum free Hb and serum CML levels (*r* = −0.3653, *p* = 0.0004) and between free Hb and sRAGE (*r* = −0.2809, *p* = 0.004). Our findings are in agreement with early in vitro studies which suggest that CML can acts as an iron chelator [23,33]. However, free Hb is pro-oxidant and metal ion is known to promote the formation of AGEs [23]. sRAGE can acts as decoy to bind AGEs and prevent the activation of AGE-RAGE pathway. The chelation of free Hb by CML may help to maintain the scavenge ability of sRAGE and prevent free Hb-mediated oxidative cascades. The animal study demonstrated that sex organs like testes appear to be prone to AGE-RAGE activation than metabolic organs like the liver as high-dose iron (>1 g ferric iron/kg diet) decreased total T levels with concomitant presence of testicular iron and AGEs-RAGE accumulation. Although >1 g ferric iron increased hepatic CML levels, no statistical difference was found in hepatic RAGE expression. This suggests that the liver was efficiently taking up and degrading AGEs-modified protein even with increased metabolic demands of metabolizing iron and fat droplets in obese animals. Overall, our animal and human studies suggested that, unlike the metabolic organ, sex organ is less equipped to handle the toxic compounds and accumulation of iron-AGEs may directly impair T biosynthesis through induction of oxidative and pro-inflammatory cytokine. 

The current study found that >1 g of ferric iron suppressed serum and testicular total T levels; however, 2 g of iron had less severe effect due to high intra-individual group variations. The concentration of total T in the blood circulation can be influenced by age, diet, obesity, and the time at which blood is withdrawn [44,45,46]. Brambilla et al. [47] suggested that intra-individual variations, and not assay variations, accounted for biological variations in the male sex hormone. In order to control diurnal variation effects on circulating total T levels [47], blood was drawn in the morning at 09:00~10:00 in all rats. The unpalatable diet of 2 g of iron plus HFD may partially explain the high intra-individual variability of the 2-g iron group. In addition, some rats may have eaten less than the others as two or three rats were kept in a single cage due to space limitations in the animal facility center. 

Our current findings support the view that AGE accumulation may exist prior to changes in systemic AGE/CML levels. In this study, we found no difference in serum CML levels, but >1 g of ferric iron caused testicle AGE accumulation, although 2 g of iron had a less-significant effect on hepatic AGE trapping. Our previous study in diabetic rats also found that high doses of ferric iron caused AGE accumulation in the liver, no changes in plasma AGEs, and decreased plasma CML levels [27]. An inverse relationship between serum AGE/CML levels and obesity was observed in several human studies [48,49,50]. Gaens et al. reported that CML accumulates in adipose tissues [48] and proposed that obesity is characterized by RAGE-mediated CML trapping, and activation of the CML-RAGE axis may result in lower circulating CML levels and higher tissue inflammation. Hence, lower levels of circulating AGEs/CML may serve as a marker of a greater accumulation or trapping of AGEs in adipose tissues [50].

It is also worthwhile to consider the body’s handling of AGEs in different organs under iron/fat-induced stress. The current study noted that sex organs like testes seemed to respond differently to iron-mediated AGE-RAGE activation than metabolic organs like the liver. Although 1 g of ferric iron increased hepatic AGE/CML levels, no statistical difference was found in hepatic RAGE expression. This suggests that the liver was efficiently taking up and degrading AGEs, even with increased metabolic demands of metabolizing iron and fat droplets in obese animals. Much to our surprise, the addition of ferric iron seemed to affect the ability of the testes to metabolize AGEs as 1 g of ferric iron induced AGE-RAGE accumulation predominately in testicular interstitial areas. RAGE is the best-characterized AGER, and ligation of AGE-RAGE triggers pro-inflammation or oxidative stress response resulting in Leydig’s cell dysfunction and a T deficiency [51].

It has long been recognized that genetic iron overload can lead to male hypogonadism [15]. A recent clinical study conducted by Mirlohi et al. reported that serum AGEs were positively correlated with iron overload and markers of oxidative stress in patients with ß-thalassemia major [37]. As far as we are aware, mechanisms underlying iron-mediated endogenous AGE formation, trapping, and clearance are still unclear. In the current iron/fat-induced obese rat model, the HFD alone induced comparable levels of MDA as did iron plus the HFD, which resulted in no significant correlation between testicular MDA and CML levels (*r* = 0.171, *p* = 0.357) (data not shown). In contrast, >1 g of iron significantly induced testicular TNF-α which was positively correlated with testicular CML (*r* = 0.484, *p* < 0.05), suggesting that iron-mediated inflammation may play a role in AGE accumulation (data not shown). Alternatively, iron/fat-mediated AGE accumulation or toxicity may be due, in part, to interference with modified AGE degradation. The current study found that, in testes, high-dose iron increased the expression of the AGE-RAGE axis and AGE uptake receptors (AGER1 and CD36), but no difference in the expression of the AGE degradation enzymes, CTSD and GLO-1. A similar finding was also observed in the liver in which hepatic AGEs/CML and AGER1 increased, but no differences were found in CD36, RAGE. 

Our study showed synergistic effects of HFD and iron on T and AGEs metabolism. When compared to those of lean rats, obese rats showed no reduction in serum and testicular total T levels in spite of marginal increases in serum and testicular CML levels. Although obese rats had slightly higher serum CML levels than those of lean rats, we found no difference in HbA1c level (lean: 4.3% vs. 4.5%). Mu et al. [52] conducted a 8 weeks HFD feeding experiment and observed that serum T levels in obese mice were initially increased then gradually decreased and the reduction of total T levels was correlated with the decreased size of testis. In our current rat experiment, the addition of 50% HFD or HFD plus ferric iron did not reduced the size of testis compared with healthy lean rats. It is likely that our experimental obese rats were healthier and had less severe testicle damage compared to those mice of Mu [52]. However, the addition of >1 g ferric iron plus HFD diet significantly decreased total T levels and this was associated with increased serum MDA levels, but not serum CML. Detail examination of liver and testicle tissues showed that >1g ferric iron results in testicular iron and AGEs/CML accumulation and this is associated with increased expression of testicular RAGE as well as oxidative (NO, MDA) and pro-inflammatory markers (TNF-α). Although an HFD alone induced comparable levels of testicular MDA as did the iron/fat-enriched diet, high doses of iron (1 g or 2 g of ferric iron) also increased expressions of testicular TNF-α and NO as well as antioxidants (e.g., SOD2 and catalase). This suggests that high-dose iron triggers inflammatory responses as well as oxidative stress in testes. Alternatively, the ligation of AGEs-RAGE mediated by high doses of iron may further contribute to activation of TNF-α and NO synthesis.

The effects of dietary lipids on the male sex hormone have been known for more than 30 years [53]. Cholesterol is the precursor of all steroid hormones including the male sex hormone, T. But a meal rich in fat (containing 1300 kcal, 11% carbohydrates, 3% protein, and 86% fat) was shown to decrease circulating total T (−22%) in 11 healthy men, and this effect lasted for 1~8 h [54]. A complex multidirectional relationship between obesity and an androgen deficiency may exist. Obesity is associated with decreased gonadotropin-releasing hormones (e.g., luteinizing hormone, LH), and low circulating LH may suppress T synthesis by affecting Leydig’s cells [51]. Obesity-related inflammation might also directly contribute to T deficiency [51]. In addition, an HFD may enhance endogenous AGE formation [55,56]. An animal study showed that an HFD induced visceral AGE formation [55], and mice fed high dietary AGEs exhibited increased systemic inflammatory cytokines and weight gain [57]. An in vitro study showed that AGEs suppressed human chorionic gonadotropin-induced T synthesis in a dose-dependent manner in primary rat Leydig’s cells via inducing oxidative stress and endoplasmic reticular stress-associated pathways [21]. Collectively, these data suggest that an HFD may interfere with the metabolism of modified AGEs, and increased testicular AGE accumulation may cause Leydig’s cell dysfunction, leading to decreased T synthesis.

## 5. Conclusions

Collectively, our results demonstrate that a high dosage of iron supplementation may affect testicular ability to degrade the modified AGE, and iron-AGE-RAGE pathways may serve as potential targets to manage low T in obese individuals with iron metabolism dysregulation.

## Figures and Tables

**Table 1:** Clinical characteristics of adult men stratified by body weight status (*n* = 111).

Variables	Body Weight Status		*p*-Value
	Normal Weight (*n* = 44)	OW/Obese (*n* = 67)	
Basic characteristic			
Age (years)	39.40 ± 12.35	42.20 ± 10.84	0.164
BMI (kg/m^2^)	21.86 ± 1.48	28.26 ± 3.65	<0.001
Hypogonadism (*n*,%)	5 (11.3)	13 (19.0)	<0.001
Iron overload (*n*,%)	7(15.9)	23 (29.8)	<0.001
AGE biomakers			
CML (μg/mL)	270.98 ± 109.28	363.18 ± 129.87	0.032
sRAGE (pg/mL)	361.58 ± 255.41	372.10 ± 190.03	0.293
CTSD (ng/mL)	2147.21 ± 1547.46	1720.48 ± 1485.99	0.200
MDA (μM)	43.11 ± 20.96	46.68 ± 27.79	0.626
Iron biomarkers			
Free Hb (μg/mL)	139.08 ± 42.42	165.70 ± 53.71	0.003
Ferritin (ng/mL)	205.53 ± 151.63	242.66 ± 156.18	0.080
Transferrin saturation (%)	34.34 ± 11.19	32.87 ± 12.60	0.300
Serum iron (μg/mL)	107.5 ±32.5	108.3 ± 37.1	0.954
RBC Aggregation CSS (mPa)	262.78 ± 63.50	286.60 ± 62.85	0.044
Sex hormone biomarkers			
Total T (ng/mL)	4.77 ± 1.30	3.62 ± 1.09	<0.001
Free T (pg/mL)	90.58 ± 26.86	79.51 ± 24.15	0.042
SHBG (nmol/L)	36.21 ± 13.55	29.79 ± 14.69	0.003

*p*-value was analyzed by Mann-Whitney U test and chi-square for categorical variables. Continuous data present as mean ± SD, categorical data present as number (percentage of same group) Iron overload was defined as adult men with serum ferritin >300 ng/mL.

**Table 2:** Spearman correlation coefficiency of total testosterone, iron and AGE biomarkers in male participants (*n* = 111).

Variables	Total T (ng/mL)		Serum CML (μg/mL)		sRAGE (pg/mL)	
	*r*	*p*-Value	*r*	*p*-Value	*r*	*p*-Value
Iron biomarkers						
Transferrin saturation (%)	0.242	0.007	−0.132	0.207	0.0214	0.832
RBC Aggregation CSS (mPa)	−0.419	<0.0001	0.0957	0.359	0.0026	0.98
Serum iron(μg/mL)	0.039	0.665	−0.0423	0.697	0.0114	0.912
Serum ferritin (ng/mL)	−0.117	0.194	0.1343	0.204	0.0559	0.578
Free Hb (μg/mL)	−0.1308	0.192	−0.3653	0.0004	−0.2809	0.004
AGE biomakers						
CML(μg/mL)	−0.209	0.030				
Cathepsin D (ng/mL)	0.3302	0.001	−0.071	0.526	0.0583	0.581
Malondialdehyde (μM)	0.026	0.818	0.1537	0.168	0.0185	0.863
sRAGE (pg/mL)	−0.1309	0.192	0.2132	0.042		
